# Clinical application of robot-assisted laparoscopic resection of large pheochromocytomas via the renal surface plane using the fatty fissure as an anatomical landmark

**DOI:** 10.12669/pjms.42.4.13369

**Published:** 2026-04

**Authors:** Deqiang Gu, Tao Ma, Wei Zhang, Yang Yu, Yong Suo, Jingyang Guo

**Affiliations:** 1 Deqiang Gu, Department of Urology, The Affiliated Hospital of Hebei University, Baoding 071000, Hebei, China; 2 Tao Ma, Department of Urology, The Affiliated Hospital of Hebei University, Baoding 071000, Hebei, China; 3 Wei Zhang, Department of Urology, The Affiliated Hospital of Hebei University, Baoding 071000, Hebei, China; 4 Yang Yu, Department of Urology, The Affiliated Hospital of Hebei University, Baoding 071000, Hebei, China; 5 Yong Suo, Department of Urology, The Affiliated Hospital of Hebei University, Baoding 071000, Hebei, China; 6 Jingyang Guo, Department of Urology, The Affiliated Hospital of Hebei University, Baoding 071000, Hebei, China

**Keywords:** Anatomical landmark, Fatty fissure, Large pheochromocytoma, Robot-assisted surgery

## Abstract

**Objective:**

To evaluate the clinical utility of robot-assisted laparoscopic resection for large pheochromocytomas via the renal surface plane, using the fatty fissure as an anatomical landmark.

**Methodology:**

This retrospective study included 60 patients with large adrenal pheochromocytomas treated at The Affiliated Hospital of Hebei University (January 2023–December 2024), randomized into two groups (n=30 each). The study group underwent the landmark-guided procedure, while the control group received conventional posterior retroperitoneal robot-assisted laparoscopic surgery. Perioperative parameters (operation duration [DoO], intraoperative blood loss [IBL], drain removal time [DRT], postoperative hospital stay [LoPHS]) and complications (intraoperative hemodynamic instability [IHDI], vasoactive agent use, ICU transfer rate, adrenal crisis, arrhythmia, stress ulcers) were compared.

**Results:**

The study group showed significantly shorter DoO, LoPHS and DRT (P=0.00), lower IBL (P=0.00), reduced IHDI incidence, vasoactive agent use and ICU transfer rate (P<0.05 each), and a lower overall postoperative complication rate (P=0.04) versus the control group.

**Conclusion:**

Fatty fissure-guided robot-assisted laparoscopic adrenalectomy via the renal surface plane improves anatomical guidance, reduces IBL, shortens operative and recovery time, and lowers complications for large pheochromocytomas, representing a safe, effective option for wider clinical use.

## INTRODUCTION

Adrenal tumors are relatively common neoplasms of the urinary system and include functional and non-functional adrenal tumors, pheochromocytomas, adrenocortical carcinomas, and adrenal metastases. Among these, pheochromocytoma is one of the most frequently encountered adrenal neuroendocrine tumors.^1^ Pheochromocytes are responsible for the synthesis and storage of catecholamines; elevated catecholamine levels in the bloodstream can lead to symptoms such as hypertension and tachycardia. When triggered by stimuli such as trauma, anxiety, surgery, or anesthesia, a sudden surge of catecholamines can be released into the circulation, potentially triggering life-threatening cardiovascular complications. Large pheochromocytomas are typically defined as tumors with a diameter greater than 6 cm, which present considerable technical challenges during surgical resection.^2^ Suzuki K et al.^3^ reported that compared with smaller tumors, laparoscopic resection of large pheochromocytomas is associated with greater hemodynamic instability and increased risk of intraoperative bleeding.

Given the malignant potential and technical complexity of large pheochromocytomas, the role of laparoscopic adrenalectomy (LA) in their management remains a subject of ongoing debate.^4^ Ou MJ et al.^5^ reported that, compared with open adrenalectomy, minimally invasive surgery for large pheochromocytomas is associated with a significantly lower complication rate (*P =* 0.04), shorter postoperative hospital stay (*P =* 0.02), and reduced intraoperative blood loss (IBL) (*P =* 0.002). However, conventional laparoscopic surgery has inherent limitations, such as limited dexterity, reliance on two-dimensional vision, and dependence on an assistant to manage the camera. Robot-assisted surgery offers the potential to overcome these challenges. Compared with standard laparoscopy, robot-assisted laparoscopic surgery provides enhanced maneuverability, more flexible instrument articulation, and a three-dimensional visual field that enables greater surgical precision.^6^ Traditionally, posterior retroperitoneal LA is performed by dissecting along the surface of the perinephric adipose capsule.^7^

However, this approach often results in the tumor being encased within the surrounding fat tissue, complicating subsequent surgical maneuvers. The extended time required to locate and mobilize the tumor may lead to repeated manipulation and inadvertent compression of pheochromocytoma tissue, causing sudden surges of catecholamines into the central venous system.^8^ This can induce dramatic fluctuations in blood pressure and heart rate. In our practice, we adopted a novel approach, *i.e*., robot-assisted laparoscopic resection of large pheochromocytomas via the renal surface plane, using the fatty fissure as a key anatomical landmark. This method provides clearer anatomical orientation and facilitates more efficient identification of the tumor and associated vasculature, demonstrating considerable clinical value. The present study reports our clinical experience and outcomes with this surgical approach.

## METHODOLOGY

This was a retrospective study. A total of 60 patients with large adrenal pheochromocytomas who were admitted to The Affiliated Hospital of Hebei University between January 2023 to December 2024 were enrolled and randomly assigned to two groups(n = 30 each). The study group underwent robot-assisted LA via the renal surface plane using the fatty fissure as an anatomical landmark. The control group underwent conventional posterior retroperitoneal robot-assisted laparoscopic adrenalectomy via the perinephric adipose capsule plane.

### Ethical approval:

The study was approved by the Institutional Ethics Committee of The Affiliated Hospital of Hebei University (No.HDFYLL-KY-2023-010; Date: January 28, 2023), and written informed consent was obtained from all participants.

### Inclusion criteria:

- Diagnosis confirmed by clinical history, imaging studies, and postoperative pathological examination.
- Age ≤ 75 years.
- Absence of major comorbidities affecting the heart, lungs, or other critical organs, and no contraindications to surgery.
- Tumor diameter > 6 cm.
- Stable blood pressure and heart rate prior to surgery.
- Complete clinical data available.
- Written informed consent obtained, with good compliance and ability to complete all study procedures.

### Exclusion criteria:

- History of prior surgery on the affected side or localized skin lesions in the surgical field.
- Severe dysfunction of major organs (e.g., heart, brain, lungs, liver, kidneys) that could not be adequately corrected preoperatively.
- Coagulation disorders.
- Uncontrolled blood pressure or heart rate.
- Psychiatric or neurological disorders impairing compliance with study protocols.
- Incomplete clinical data.

In the study group, patient age ranged from 42 to 74 years (mean: 58.37 ± 16.23 years), while in the control group, age ranged from 44 to 73 years (mean: 57.82 ± 16.50 years). There were no statistically significant differences in baseline characteristics between the two groups (all *P >* 0.05), indicating good comparability (Table-I).

**Table-I:** Comparison of baseline characteristics between the two groups (*x̅*+*s*, n = 30 per group).

Item	Study group	Control group	t/χ^2^ value	P-value
Age	58.37±16.23	57.82±16.50	0.32	0.64
Tumor location			0.43	0.67
Left	17(57%)	19(63%)		
Right	13(43%)	11(37%)		
Tumor diameter (cm)	6.76±0.38	6.83±0.45	0.65	0.52
***Associated symptoms***				
Hypertension	16(53%)	18(60%)	0.27	0.60
Palpitations	6(20%)	5(17%)	0.11	0.74
Hypokalemia	4(13%)	7(23%)	0.83	0.32

P > 0.05.

### Surgical Methods:

In the study group, patients underwent robot-assisted LA via the renal surface plane using the fatty fissure as an anatomical landmark. Under general anesthesia, the retroperitoneal space was established, and robotic arms along with the laparoscopic camera were positioned. Extraperitoneal fat was first cleared, from the subdiaphragmatic region down to the iliac fossa. The lateroconal fascia was incised near the peritoneal reflection, and perirenal fat was incised along the peritoneal side down to the surface of the renal parenchyma. Blunt and sharp dissection was carried out along this plane to mobilize the kidney surface.

The upper pole of the kidney was completely freed from surrounding tissues and displaced posteroinferiorly, allowing separation of the upper renal pole from the tumor and full exposure of the adrenal region. At this stage, the adrenal tumor and its surrounding fat were suspended above the superomedial aspect of the kidney. A distinct elliptical fissure-like structure formed between the peritumoral fat, adrenal fat, and perirenal fat. The larger the tumor, the more prominent this fissure becomes due to the mass effect. Dissection proceeded along this fissure, separating peritumoral fat from perirenal fat, ventrally toward the peritoneum and dorsally toward the psoas major muscle. These two planes were expanded and merged at the tumor base. At this point, the tumor was nearly completely mobilized. If normal adrenal tissue was minimal or difficult to distinguish, the entire adrenal gland and tumor were excised en bloc. When feasible, partial adrenal preservation was attempted, excising the tumor with a small margin of adjacent adrenal tissue.

In the control group, patients underwent conventional posterior retroperitoneal robot-assisted LA. The procedure began with the clearance of extraperitoneal fat. The lateroconal fascia was opened near the peritoneal reflection, followed by sequential dissection along three anatomical planes on the surface of the perinephric adipose capsule:

- the prerenal retroperitoneal space,
- the posterior pararenal space anterior to the psoas major muscle, and
- the space between the kidney and adrenal gland. Fat over the middle and upper poles of the kidney was removed to expose the adrenal region. The tumor was then located and excised in its entirety.

### Outcome Measures:

- ***Surgical Parameters:*** Comparative analysis was conducted between the two groups in terms of duration of operation (DoO, measured from the start of extraperitoneal fat clearance to tumor resection), intraoperative blood loss (IBL), drain removal time (DRT), and length of postoperative hospital stay (LoPHS).
- ***Intraoperative Pheochromocytoma-related Complications:*** The incidence of intraoperative hemodynamic instability (IHDI), the proportion of patients requiring vasoactive agents, and the postoperative ICU transfer rate were compared between the two groups. IHDI was defined as systolic blood pressure >180 mmHg or mean arterial pressure <60 mmHg.^8^
- ***Postoperative Complications:*** Postoperative outcomes were assessed and compared between the two groups, including adrenal crisis, arrhythmias, infections, and stress ulcers.

### Statistical analysis:

All data were analyzed using SPSS 20.0. Continuous variables were expressed as mean ± standard deviation (*x̅*+*s*). Between-group comparisons of continuous data were performed using independent sample t-tests, while categorical variables were analyzed using chi-square (χ^2^) tests. A *P*-value of <0.05 was considered statistically significant.

## RESULTS

The study group showed significantly shorter DoO, LoPHS, and DRT compared with the control group (*P =* 0.00, respectively). IBL was also significantly lower in the study group (*P =* 0.00) (Table-II).

**Table-II:** Comparison of surgical parameters between groups (**x̅*+*s**, *n =* 30 per group).

Group	DoO (min)	IBL (mL)	LoPHS (d)	DRT (min)
Study group	23.62±6.83	52.67±22.56	5.50±1.50	25.59±3.81
Control group	42.06±10.41	74.81±21.42	7.62±1.57	34.62±9.83
*t-value*	8.11	3.92	5.35	4.69
*P-value*	0.00	0.00	0.00	0.00

P < 0.05.

The study group demonstrated significantly lower rates of IHDI, use of vasoactive agents, and postoperative ICU transfers compared with the control group (*P <* 0.05, respectively) (Table-III). One patient in the study group developed an adrenal crisis, while the control group reported three cases of adrenal crisis, one case of arrhythmia, and two cases of stress ulcer. The overall incidence of postoperative complications was significantly lower in the study group than in the control group (*P =* 0.04) (Table-IV).

**Table-III:** Comparison of pheochromocytoma-related complications between groups (*x̅*+*s*, n = 30 per group).

Group	IHDI	Use of vasoactive agents	Postoperative ICU transfer
Study group	4(13%)	2(6%)	0(0%)
Control group	12(40%)	8(27%)	4(13%)
*χ* ^2^	5.45	4.32	4.29
*P-value*	0.02	0.04	0.04

P < 0.05.

**Table-IV:** Comparison of postoperative complications between groups (*x̅*+*s*, n = 30 per group).

Group	Adrenal crisis	Arrhythmia	Stress ulcer	Incidence (%)*
Study group	1	0	0	1(3%)
Control group	3	1	2	6(20%)
*χ* ^2^				4.04
P-value				0.04

* P < 0.05.

## DISCUSSION

The present study demonstrated that robot-assisted laparoscopic adrenalectomy (LA) via the renal surface plane using the fatty fissure as an anatomical landmark yielded significant advantages in the surgical management of large pheochromocytomas (tumor diameter > 6 cm) compared with conventional posterior retroperitoneal robot-assisted LA. Specifically, the study group exhibited remarkably shorter operation duration(DoO), postoperative hospital stay (LoPHS), and drain removal time (DRT) (all P = 0.00), reduced intraoperative blood loss (IBL) (P = 0.00), and lower incidences of intraoperative hemodynamic instability (IHDI), vasoactive agent use, ICU transfer rate, and overall postoperative complications(all P< 0.05). These findings confirm that the fatty fissure-guided renal surface plane approach enhances anatomical orientation, optimizes surgical efficiency, and improves perioperative safety for large pheochromocytoma resection.

With the continuous advancement of laparoscopic techniques in urology, LA has gradually become the preferred surgical approach for most adrenal tumors, particularly benign lesions. Although both minimally invasive procedures and open surgeries show comparable efficacy in managing hypertension and hypokalemia in adrenal tumors, the minimally invasive approach is associated with fewer postoperative complications and shorter hospital stays.^9^ Consistent with this, our study further supports the superiority of minimally invasive surgery in large pheochromocytoma management, as the study group achieved reduced IBL and shorter LoPHS compared with the conventional minimally invasive approach. Surgical resection of adrenal pheochromocytomas remains technically challenging due to the tumor’s rich vascularity and the propensity for sudden catecholamine release, which can cause dramatic intraoperative blood pressure fluctuations.

Despite improvements in surgical technique and perioperative management, IHDI remains a serious concern, potentially leading to fatal complications. Therefore, performing minimally invasive procedures for large pheochromocytomas is still considered a surgical challenge.^10^ Nevertheless, compared with open surgery, laparoscopic resection of pheochromocytomas continues to offer advantages such as reduced IBL and shorter LoPHS, which is in line with the findings of Ou MJ et al,^5^ who reported that minimally invasive surgery for large pheochromocytomas was associated with significantly lower complication rates (P = 0.04), shorter LoPHS (P = 0.02), and less IBL (P = 0.002) than open adrenalectomy. Our results extend this evidence by showing that even among minimally invasive approaches, the fatty fissure-guided technique further mitigates surgical risks and improves outcomes.

Since the first report by Yu DY et al. in 2001 on robot-assisted LA using the da Vinci system,^11^ this approach has provided a precise and reliable surgical method for adrenal tumor resection. Compared with conventional laparoscopic surgery, robot-assisted LA is similarly safe and effective and may offer distinct advantages in managing complex adrenal tumors. Studies have shown that while robot-assisted LA has comparable DoO and conversion-to-open surgery rates with conventional LA, it may offer potential benefits, such as shorter LoPHS, reduced IBL, and fewer postoperative complications.^12^ Xue Y et al. also confirmed that robot-assisted adrenalectomy for pheochromocytomas achieved favorable perioperative outcomes compared with laparoscopic adrenalectomy.^13^

In our study, both groups underwent robot-assisted surgery, but the study group still outperformed the control group in key parameters, indicating that the anatomical landmark-guided approach enhances the inherent advantages of robotic surgery. This suggests that optimizing surgical anatomy identification can further improve the efficacy of robot-assisted procedures, especially for large and complex tumors.

LA can be performed via two main approaches: transperitoneal and retroperitoneal. The retroperitoneal approach has both notable advantages and limitations. One key benefit is that it avoids entering the peritoneal cavity, thereby minimizing disturbance to intraperitoneal organs and facilitating faster postoperative recovery;^14^ most patients can resume a liquid diet by postoperative day two. This approach is also more familiar to most urological surgeons. However, it presents significant challenges, particularly due to the abundance of retroperitoneal fat and the lack of clear anatomical landmarks. This is especially problematic in obese patients or those with granular fat, where the difficulty of dissection increases substantially.

Furthermore, patients with diabetes may have inflammatory or degenerative changes in retroperitoneal fat, further complicating surgical manipulation.^15^ Conventional posterior retroperitoneal LA using three-plane dissection presents several limitations. This technique involves mobilizing the anterior and posterior perirenal spaces from outside the perirenal fat and then identifying the adrenal gland at the upper pole of the kidney. However, once the perirenal fat is dissected, the absence of tension allows it to accumulate locally, which can significantly hinder subsequent dissection. This not only increases the difficulty of locating the adrenal gland but also necessitates repeated probing and manipulation. Such maneuvers can compress the tumor, triggering catecholamine release into the bloodstream, an especially pronounced issue in obese patients.

Ma T et al.^16^ reported that adrenal depth from the skin surface is an independent predictor of DoO. Given that the adrenal glands are concealed beneath the ribs and lie in a deep anatomical position, the restricted working space in the retroperitoneal approach further compounds these technical challenges. In contrast, our study’s renal surface plane approach addresses these limitations by utilizing the fatty fissure as a clear anatomical landmark. This is consistent with Mora Fritis C et al.^9^, who reported that laparoscopic anatomical adrenalectomy via the renal cortex surface monolayer improved surgical visualization and reduced operative complexity. The fatty fissure, an avascular plane formed by the mass effect of large tumors, provides a natural dissection corridor that minimizes unnecessary manipulation and tumor compression, thereby reducing catecholamine surges and IHDI. This explains why the study group had a significantly lower incidence of IHDI (13% vs. 40%, P = 0.02) and vasoactive agent use (6% vs. 27%, P = 0.04) compared with the control group.

A tumor diameter of 6 cm is widely recognized as the threshold distinguishing small from large adrenal tumors. In patients with large adrenal tumors, the risk of intraoperative capsule rupture, significant hemorrhage, and local recurrence is notably higher than in those with smaller lesions. Traditionally, it was believed that large adrenal tumors necessitated open surgical resection.^17^ However, an increasing number of studies support the view that minimally invasive surgery is a safe and effective alternative to open surgery, even for adrenal masses exceeding 6 cm in diameter. Rossi L et al.^18^ conducted a propensity score-matched study and found that minimally invasive adrenalectomy achieved equivalent perioperative outcomes to open adrenalectomy for tumors larger than 6 cm.

Our study further strengthens this argument by showing that a refined minimally invasive approach (fatty fissure-guided robot-assisted LA) can achieve superior outcomes compared with conventional minimally invasive techniques for large pheochromocytomas. Nevertheless, surgery for large adrenal tumors is frequently accompanied by one or more complications, such as hypertensive crises and IHDI, both of which can be life-threatening if not managed promptly and appropriately.^19^,^20^ Tan Q et al.^20^ reported that transperitoneal laparoscopic adrenalectomy for large pheochromocytomas reduced IBL and LoPHS but still carried a considerable risk of intraoperative complications. In our study, the overall postoperative complication rate was only 3% in the study group, significantly lower than the 20% in the control group (P = 0.04), which may be attributed to the clearer anatomical guidance and reduced tumor manipulation afforded by the fatty fissure landmark. This suggests that identifying and utilizing natural anatomical planes is crucial for mitigating the risks associated with large pheochromocytoma resection.

Notably, some studies have reported conflicting results regarding the advantages of robot-assisted or anatomical landmark-guided approaches. For example, Zhou Y et al.^12^ conducted a meta-analysis and found that robot-assisted LA had comparable DoO to conventional LA, which differs from our finding of a significantly shorter DoO in the study group. This discrepancy may be due to differences in surgical approaches and landmark utilization. Another potential reason is the patient population: our study exclusively included large pheochromocytomas (> 6 cm), where the fatty fissure landmark is more prominent due to the mass effect, thereby maximizing the benefits of the approach. In smaller tumors, the fatty fissure may be less distinct, limiting the advantage of this technique. Additionally, regional differences in surgical expertise and perioperative management may contribute to varying outcomes. For instance, urological surgeons in our institution have extensive experience with retroperitoneal robot-assisted surgery, which may have optimized the implementation of the fatty fissure-guided approach.

Another point of consideration is the choice between retroperitoneal and transperitoneal approaches. Haskins L et al.^14^ reported that robotic-assisted retroperitoneal laparoscopy for renal tumors achieved faster recovery than traditional retroperitoneal laparoscopy, which aligns with our study’s finding of shorter LoPHS in the study group. However, the transperitoneal approach may offer a larger working space for very large tumors, which could be a limitation of the retroperitoneal approach adopted in our study. Future studies could compare the fatty fissure-guided technique with transperitoneal robot-assisted LA for tumors exceeding 8 cm to further clarify the optimal approach for extremely large pheochromocytomas.

### Limitations:

It includes the modest sample size and the lack of long-term follow-up data. In future work, we plan to expand the sample size and extend the duration of postoperative follow-up to more objectively assess the strengths and limitations of this technique, aiming to optimize outcomes and benefit a wider patient population.

## CONCLUSIONS

Robot-assisted LA via the renal surface plane, using the fatty fissure as a key anatomical landmark, offers distinct advantages in the surgical management of large adrenal pheochromocytomas. This approach can facilitate clearer anatomical dissection, reduce IBL, shorten DoO, promote postoperative recovery, and decrease complication rates, representing a promising surgical strategy worthy of broader clinical application.

### Authors’ Contributions:

**DG** and **TM:** Literature search**,** Conceived, designed, data analysis, write up, supervised and final approval of the manuscript.

**WZ** and **YY:** Data collection, data analysis, Write up.

**YS:** Designed, data analysis and write up.

**JG:** responsible and accountable for the accuracy/integrity of the work.

All authors have read and approved the final manuscript.
